# Septic arthritis in patients with rheumatoid arthritis

**DOI:** 10.1186/1749-799X-3-33

**Published:** 2008-07-29

**Authors:** Abdulaziz Al-Ahaideb

**Affiliations:** 1 College of medicine, King Saud University, Riyadh, Saudi Arabia

## Abstract

There is an increasing number of rheumatoid patients who get septic arthritis. Chronic use of steroids is one of the important predisposing factors. The clinical picture of septic arthritis is different in immunocompromised patients like patients with rheumatoid arthritis. The diagnosis and management are discussed in this review article.

## Introduction

The association of septic arthritis in patients with rheumatoid arthritis has been recognized for over fifty years [1]. Since that time there have been over 400 reported cases in the literature [2,3] but to date no mechanism for this increased susceptibility has been confirmed. Diagnosis of septic arthritis in the rheumatoid patient is often delayed with a notably worse outcome when compared to other patients with septic arthritis [4]. Little has been published on the topic of septic arthritis in patients with rheumatoid arthritis. Work by Goldenberg [5] in 1989 and Gardner and Weisman [6] in 1990 where they presented their case experience and reviewed the literature, is still considered the standard when discussing this topic.

## Discussion

### Pathogenesis

Any chronically arthritic joint is predisposed to infection [5]. The mechanisms responsible for this have yet to be precisely identified but previously published data [3,5] have highlighted some of the possible contributing factors. It has been hypothesized that in the rheumatoid patient that phagocytosis by the polymorphonuclear (PMN) cells in the blood and synovial fluid is defective [7,8] leading to the increased susceptibility to infection. Turner et al. [7] thought that this was likely due to increased ingestion of immune complexes by the PMN's in the synovial fluid leading to impaired uptake. While Wilton et al. [8] postulated that this decreased ability to ingest and kill bacteria could be due to deficiency in the expression of C'3 on the PMN in the synovial fluid. However, more recently, Breeveld et al [9] failed to observe any defect in phagocytosis by the PMN's. Their work demonstrated that both uptake and intracellular killing of *Staphylococcus aureus *was intact in the synovial fluid and peripheral blood.

Abnormal joint structure and pre-existing joint lesions have also been implicated in the increase susceptibility of the rheumatoid patient to septic arthritis. It is thought that anomalous joint structure present in rheumatoid patients could allow microorganisms to escape normal phagocytosis [5]. Mahowald et al. [10], using the Dumonde Glynn model of antigen induced arthritis in rabbits followed by introduction of *S. aureus*, hypothesized that infection in the arthritic joint extends along the pannus to the subchondral bone. There is extensive neovascularization in the subsynovium in an arthritic joint. She postulated that the vascualrization subsequently becomes occluded with bacteria leading to the ischemic changes of the subchondral bone and subsynovium [5,10]. All of these factors combine to cause the more rapid histological changes observed in the arthritic joint when infected. It is also thought possible that microorganisms could traffic from skin lesions to draining lymph nodes through the inflamed synovium [3] or that infection could track to the joint from an adjacent focus of osteomyelitis [11].

It is known that there is a higher incidence of infection in those individuals ingesting exogenous steroids [5], although 50% of patients with rheumatoid arthritis who developed polyarticular septic arthritis (PASA) were not receiving steroids [3]. It is therefore thought that there is also a general reduction in resistance to infection in the rheumatoid patient. This leads to a higher incidence of systemic complications in this patient population [12]. In fact, Vandenbroucke et al. [13] observed that bronchitis and pneumonitis were more severe in the rheumatoid patient when compared to a patient with osteoarthritis. It was however noted that there was no difference in the frequency of infection between these two populations and there was also little difference in the type of infection present [14].

Ostensson and Genborek [15] also stressed the important role of previous intra-articular injections even in the development of septic arthritis months following injection. There have been some reports of isolated glucocorticoid injection resulting in infection [16,17] but the rate observed by this group was uniquely elevated [16].

Tumor necrosis factor alpha (TNF-alpha) plays an important role in the host defense against infection. Inhibition of its activity could therefore be anticipated to augment the risk of infection. A marked increase in opportunistic infections, particularly tuberculosis, has been described with etanercept, an agent that blocks TNF-alpha activity [18,19].

### Clinical Features

Typically a patient with septic arthritis presents with acute onset of severe joint pain, exacerbated with minimal movement, accompanied by swelling and erythema at the effected joint. There are also systemic manifestations of infection including fever, elevated white blood cell (WBC) count and increased erythrocyte sedimentation rate (ESR). However this is not always the case in rheumatoid patients who present with polyarticular septic arthritis (PASA). Often the onset is more insidious and is mistaken for a flare of rheumatoid arthritis.

Data compiled by Dubost et al [3] demonstrated that the average age of rheumatoid patients who developed PASA was 62 years of age and the risk of developing PASA is twice as high in males [3]. The patient's rheumatoid arthritis was most often present for more than 10 years and the patients had advanced erosive, seropositive rheumatoid arthritis [16]. Twenty percent of patients were afebrile on presentation and only 63% of patients with PASA developed a temperature above 38°C. Infection involved a mean of 3.5 joints with the knee being most common followed by elbow, wrist ankle, hip and shoulder [6]. Infections in the metatarsalphalangeal, sternoclavicular, and metacarpophalangeal joints have also been reported [6]. Individuals with hip and knee prosthesis are also at increased risk for developing septic arthritis [17].

Lab tests in these patients revealed that leukocytosis was present in 60–63% of published cases of patients with PASA. The ESR was on the average 90 mm/hr with no difference observed when comparing rheumatoid with non-rheumatoid groups. A value of >100 mm/hr was present in 49% of those with PASA [3,20,21]. Joint aspirate of synovial fluid revealed an average of 120 000 leukocytes/mm^3^. It is important to note that pyathrosis in these patients often leads to a flare of rheumatic arthritis and the individuals may have other joints where "sterile" synovial fluid is present [3]. Blood cultures were positive in 77–86% of cases published [3].

Because of the previous history of rheumatoid arthritis, the insidious onset of the symptoms and the presence of some "sterile" joints, there is often a delay in the diagnosis of the septic arthritis in these patients. In fact Blackburn et al [22] reported an average delay in diagnosis of 13.7 days and the diagnosis was often made serendipitously during an arthrogram or an intra-articular injection [5]. Microscopic analysis and culture of synovial fluid are fundamental diagnostic tools in the evaluation of possible joint sepsis. Sonographic guidance of arthrocentesis led to successful aspiration of difficult-to-access joints as shoulder and hip [23]. MR imaging is a very useful tool in diagnosing septic arthritis. The inherent tissue contrast provided by MR imaging allows for the delineation of soft-tissue infection and osteomyelitis [24].

### Bacteriology and Source of Infection

Literature reports involving patients with PASA and rheumatoid arthritis reveals that as many as 93% of the infections were caused by *S. aureus*. Other species of microorganism are poorly reported in the literature but cases of PASA caused by *Streptococcus pneumoniae*, Groups B, C and G *Strep, Hemophilus *and Gram-negative bacilli have also been reported [3,6].

The most common source of the septic arthritis was the skin. These accounted for 76% of the cases where a source of infection could be identified [6]. Often these were rheumatoid nodules or ulcerated calluses of the rheumatoid foot [3,6]. Other identified sources of infection include urinary tract, lung, and GI tract [6].

### Management and Outcome

Once the diagnosis of septic arthritis is suspected, joint aspirate should be performed with initial choice of antibiotic based on Gram stain [5,16]. Gram positive cocci can be treated with vancomycin or a third generation cephalosporin while Gram negative bacilli are best treated with a third generation cephalosporin in addition to an aminoglycoside [5]. If the Gram stain comes back negative broad-spectrum therapy should be initiated [16].

There is little published data with respect to the optimal method of drainage of the infected joint. There are no prospective studies comparing surgical drainage and repeated needle aspiration and each case must be dealt with on an individual basis [5]. It is thought that surgical drainage may provide the patient with increased joint protection and is often the preferred mode of treatment in the patient with rheumatoid arthritis due to their increased susceptibility to joint damage. It is thought that this more aggressive approach may reduce recurrence and lower mortality [6]. Arthroscopy, however, has shown promise in some instances of pyarthrosis [25].

Mortality in rheumatic patients with PASA is as high as 50% [3,5,6]. This is significant especially when compared to rheumatic arthritis patients with monoarticular pyarthrosis whose death rate is 15%. Morbidity is also greatly affected in these patients. Goldenberg [5] reported that joint outcome is poor in rheumatoid arthritis patients compared to non-rheumatoid patients. In addition patients with rheumatoid arthritis are more likely to have a recurrence of disease when compared to those without rheumatoid arthritis [6]. It is also important to note that patients who had their treatment initiated within 7 days had the best chance at a positive outcome [6].

## Conclusion

The association of septic arthritis in the patient with rheumatoid arthritis has been recognized for several decades but in most cases it is a condition which is difficult to identify, often requiring a high degree of clinical suspicion. It is very crucial to reach the diagnosis of septic arthritis in an early stage in any case but in particular, in immuno-compromised patients like the rheumatoid patients. Late diagnosis may lead to disastrous sequale.

## Competing interests

The author declares that they have no competing interests.

## References

[B1] Kellgren JH, Ball J, Fairbrother RW, Barnes KL (1958). Suppurative arthritis complicating rheumatoid arthritis. Br Med J.

[B2] Epstein JH, Zimmerman B, Ho G (1986). Polyarticular septic arthritis. J rheumatol.

[B3] Dubost JJ, Fis I, Lopitaux R, Soubrier M, Ristori JM, Bussiere JL, Sirot J, sauvezie (1993). Polyarticular Septic Arthritis. Medicin.

[B4] Goldenberg DL, Red JI (1985). Bacterial Arthritis. N Engl J Med.

[B5] Goldenberg DL (1989). Infectious arthritis complicating rheumatoid arthritis and other chronic rheumatic disorders. Arthritis Rheum.

[B6] Gardner GC, Weisman MH (1990). Pyarthrosis in patients with rheumatoid arthritis: A report of 13 cases and a review on the literature from the past 40 years. Am J Med.

[B7] Turner RA, Schumacher HR, Myers AR (1973). Phagocytic function of polymorphonuclear leukocytes in rheumatic disease. J Clin Invest.

[B8] Wilton JMA, Gibson T, Chuck CM (1978). Defective phagocytosis by synovial fluid and blood polymorphonuclear leukocytes in patients with rheumatoid arthritis. Rheumatol Rehabil.

[B9] Breedveld FC, LaFeber GJM, Barselaar MT van den, van Dissel JT, Leijh PCJ (1986). Phagocytosis and intracellular killing of Staphylococcus aureus by polymorophonuclear cells from synovial fluid of patients with rheumatoid arthritis. Arthrits Rheum.

[B10] Mohowald ML (1986). Animal models of infectious arthritis. Clin Rheum Dis.

[B11] Atcheson SG, Ward JR (1978). Acute hematogenous osteomyelitis progressing to septic synovitis and eventual pyarthrosis: the vascular pathway. Arthritis Rheum.

[B12] Baum J (1971). Infection and rheumatoid arthritis. Arthritis Rheum.

[B13] Vanderbroucke JP, Kaaks R, Valkenburg HA, Boersma JW, Cats A, Festen JJM, Hartman AP, Huber-Bruning O, Rasker JJ, Weber J (1987). Frequency of infections among rheumatoid arthritis patients, before and after disease onset. Arthritis Rheum.

[B14] Van Albada-Kuipers GA, Linthorst J, Peeters EAJ, Breeveld FC, Dijkmans BAC, Hermans J, vandenbroucke JP, Cats A (1988). Frequency of infection among patients with rheumatoid arthritis versus patients with osteoarthritis or soft tissue rheumatism. Arthritis Rheum.

[B15] Ostensson A, Geborek P (1991). Septic arthritis as a non-surgical complication in rheumatoid arthritis: Relation to disease severity and therapy. Br J Rheumatol.

[B16] Nolla JM, Gomez-Vaquero C, Fiter J, Mateo L, Juanola X, Rodriguez-Moreno J (2000). Pyarthrosis in patients with rheumatoid arthritis: A detailed analysis of 10 cases and literature review. Semin Arthritis Rheum.

[B17] Kaandorp CJE, van Schaardenburg D, Krijnen P, Habbema JDF, Laar MAFJ ven de (1995). Risk factor for septic arthritis in patients with joint disease. Arthritis Rheum.

[B18] Cunnane G, Doran M, Bresnihan B (2003). Infection and biological therapy in rheumatoid arthritis. Best Pract Res Clin Rheumatol.

[B19] Mor A, Mitnick HJ, Greene JB, Azar N, Budnah R, Fetto J (2006). Relapsing oligoarticular septic arthritis during etanercept treatment of rheumatoid arthritis. J Clin Rheumatol.

[B20] Kaandorp CJE, van Schaardenburg D, Krijnen P, Habbema JDF, Laar MAFJ ven de (1995). Risk factor for septic arthritisin patients with joint disease. Arthritis Rheum.

[B21] Travers V, Norotte G, Roger B, Apoil A (1988). Traitement des arthrites aigues a pyogenes des grosses articulations des membres. A propos de 79 cas. Rev Rhum.

[B22] Blackburn WD, Dunn TL, Alarcon GS (1986). Infection versus disease activity in rheumatoid arthritis: eight years' experience. South Med J.

[B23] Schiavon F, Favero M, Carraro V, Riato L (2008). Septic arthritis: what is the role for the rheumatologist?. Reumatismo.

[B24] Bancroft LW (2007). MR imaging of infectious processes of the knee. Radiol Clin North Am.

[B25] Parisien JS, Shaffer B (1992). Arthroscopic management of pyarthrosis. Clin Orthop Relat Res.

